# Supplementary material for: Hepatic Mitochondrial Dysfunction and Risk of Liver Disease in an Ovine Model of “PCOS Males”
Source: Biomedicines. 2022 May 31;10(6):1291. doi: 10.3390/biomedicines10061291 (PMC9220073; doi:10.3390/biomedicines10061291)
Supplement: Supplementary file 1 [file biomedicines-10-01291-s001.zip › biomedicines-1733316-supplementary.pdf]

Supplementary Tables

Table S1. Forward and reverse primer sequences for genes analysed in the fetal and postnatal hepatic samples using qPCR and qRT-PCR.

Gene	Forward Sequence	Reverse Sequence
MT-ND1	CAACGTTCTAACACTCATCATTCC	GGGTCGTAGGGGTTCTTTGA
MT-ND2	AATTGCCCACATAGGCTGAA	GGGGGAAGTCCTCCTATTGA
MT-ND6	GGGGGTATGATGGTAGTTTTTG	AACCCAGAATCCCCCGTAT
RPS11	ATCCGCCGAGACTACCTTCA	CCTGAAGCAGGGAGAAAGGT
RPS25	CCTCACCCCAGGACAGTTTC	CAGCATGGGGAAAGTGGGAT
NDUFA7	CGCCCTCGATTGTCATGTCT	CCCCCAGCCAAAGTCATAGG
NDUFA12	TCTGGGATGTGGATGGAAGC	TACGTACTGTTGTGGAGTGCC
NDUFB4	GAAGATCCTGCCTTGATTCG	CAATTCCGAACAGAGCTCCTA
CYCS	GGTGATGTTGAGAAAGGCAAG	TTGTTTTTGTTGGCATCTGTG
UQCRB	AACGCTTCTGTGTCAGGTCA	AACCCGGCAGCGTTGTAATA
UQCRC1	AAAACCTCCTCCGAAACGCT	CCGTATGTCAGGAGACTGCG
COX5A	CGCTGGGTGACATACTTCAA	GATGCGAACTGCACTAGCAA
COX6B1	CCAGACTGCTCCTTTTGACA	GATATGGGGCAAAGGGACTT
COX7B	AGATTGGCATACAGTGGGGC	TGAGCAGCAGAGTGCTTAAAGA
ATP5IF1	CTCGGAATCGGGAGATAGTG	GGAAGTATCGCTCCTCTTCG
ATP5MC3	GCTGTCCCGAAGAGTGCAAG	CGCAGTGGTCTGAAACTCCC
ATP5PO	GACTCGGGTTTGACCTACAG	AAGAGCTGAGGCATAGCGAC

Table S2. Differentially expressed hepatic genes and proteins relevant to TCA cycle and OXPHOS in adolescent prenatally androgenized males as compared with adolescent male controls.

Gene ID	Template	Detection technique	Fold change	P Value	Q Value
**TCA cycle**					
CS	mRNA	RNAseq	-1.43	0.00	0.00
CS	protein	Proteomics	-1.12	0.06	0.35
ACO2	protein	Proteomics	-1.37	0.01	0.14
IDH2	mRNA	RNAseq	-1.29	0.01	0.07
IDH2	protein	Proteomics	-1.13	0.14	0.49
IDH3A	mRNA	RNAseq	-1.15	0.04	0.20
OGDH	mRNA	RNAseq	-1.16	0.00	0.05
OGDH	protein	Proteomics	-1.24	0.00	0.14
SUCLG1	mRNA	RNAseq	-1.13	0.00	0.04
SUCLA2	mRNA	RNAseq	-1.14	0.03	0.17
SUCLA2	protein	Proteomics	-1.51	0.18	0.53
SDHA	mRNA	RNAseq	-1.03	0.59	0.80
SDHA	protein	Proteomics	-1.62	0.02	0.22
SDHB	mRNA	RNAseq	-1.12	0.33	0.61
SDHB	protein	Proteomics	-1.17	0.02	0.24
SDHC	mRNA	RNAseq	-1.09	0.08	0.28
SDHC	protein	Proteomics	-1.31	0.38	0.72
SDHD	mRNA	RNAseq	-1.11	0.01	0.10
MDH2	mRNA	RNAseq	-1.09	0.04	0.19
MDH2	protein	Proteomics	-1.15	0.04	0.32

**OXPHOS Complex I**					
NDUFA1	mRNA	RNAseq	-1.11	0.03	0.17
NDUFA12	mRNA	RNAseq	-1.19	0.00	0.01
NDUFA2	mRNA	RNAseq	-1.13	0.02	0.14
NDUFA2	protein	Proteomics	-1.18	0.22	0.58
NDUFA4	mRNA	RNAseq	-1.20	0.00	0.03
NDUFA4	protein	Proteomics	-1.14	0.08	0.42
NDUFA5	mRNA	RNAseq	-1.27	0.00	0.02
NDUFA7	mRNA	RNAseq	-1.25	0.00	0.03
NDUFA7	protein	Proteomics	-1.81	0.03	0.28
NDUFA9	mRNA	RNAseq	-1.10	0.01	0.07
NDUFA9	protein	Proteomics	-1.20	0.02	0.22
NDUFAB1	mRNA	RNAseq	-1.19	0.00	0.01
NDUFB1	mRNA	RNAseq	-1.14	0.01	0.09
NDUFB10	mRNA	RNAseq	-1.12	0.02	0.14
NDUFB11	mRNA	RNAseq	-1.13	0.01	0.09
NDUFB3	mRNA	RNAseq	-1.22	0.00	0.01
NDUFB4	mRNA	RNAseq	-1.21	0.00	0.00
NDUFB5	mRNA	RNAseq	-1.16	0.00	0.02
NDUFB6	mRNA	RNAseq	-1.14	0.01	0.08
NDUFB8	mRNA	RNAseq	-1.11	0.01	0.07
NDUFB9	mRNA	RNAseq	-1.15	0.00	0.04
NDUFB9	protein	Proteomics	-1.15	0.05	0.34
NDUFC1	mRNA	RNAseq	-1.11	0.01	0.10
NDUFC2	mRNA	RNAseq	-1.13	0.02	0.12
NDUFS3	mRNA	RNAseq	-1.17	0.00	0.01
NDUFS4	mRNA	RNAseq	-1.16	0.00	0.03
NDUFS7	mRNA	RNAseq	-1.13	0.03	0.16
NDUFS8	mRNA	RNAseq	-1.15	0.00	0.03
NDUFS8	protein	Proteomics	-1.15	0.03	0.28
NDUFV2	mRNA	RNAseq	-1.24	0.00	0.00
**OXPHOS Complex III**					
CYC1	mRNA	RNAseq	-1.16	0.01	0.07
CYC1	protein	Proteomics	-1.25	0.01	0.14
CYCS	mRNA	RNAseq	-1.23	0.00	0.05
UQCRB	mRNA	RNAseq	-1.26	0.00	0.00
UQCRC1	mRNA	RNAseq	-1.15	0.01	0.07
UQCRC2	mRNA	RNAseq	-1.12	0.01	0.10
UQCRC2	protein	Proteomics	-1.14	0.05	0.33
UQCRFS1	mRNA	RNAseq	-1.12	0.02	0.13
UQCRH	mRNA	RNAseq	-1.20	0.00	0.01
**OXPHOS Complex IV**					
MT-CO1	mRNA	RNAseq	-1.15	0.03	0.17
MT-CO2	mRNA	RNAseq	-1.20	0.01	0.09
MT-CO2	protein	Proteomics	-1.38	0.10	0.44
COX4I1	mRNA	RNAseq	-1.08	0.03	0.17
COX5A	mRNA	RNAseq	-1.30	0.00	0.00
COX5B	mRNA	RNAseq	-1.14	0.00	0.06
COX6B1	mRNA	RNAseq	-1.12	0.00	0.05
COX6C	mRNA	RNAseq	-1.15	0.00	0.03
COX7A1	mRNA	RNAseq	-1.64	0.02	0.13
COX7A2	mRNA	RNAseq	-1.12	0.02	0.14
COX7B	mRNA	RNAseq	-1.19	0.00	0.03

OXPHOS Complex V					
MT-ATP8	mRNA	RNAseq	-1.18	0.03	0.16
MT-ATP8	protein	Proteomics	-1.23	0.57	0.83
ATP5F1A	mRNA	RNAseq	-1.11	0.03	0.17
ATP5F1A	protein	Proteomics	-1.10	0.11	0.45
ATP5F1B	mRNA	RNAseq	-1.12	0.01	0.07
ATP5F1C	mRNA	RNAseq	-1.15	0.00	0.04
ATP5F1E	mRNA	RNAseq	-1.12	0.02	0.13
ATP5F1E	protein	Proteomics	-1.14	0.37	0.71
ATP5IF1	mRNA	RNAseq	-1.17	0.00	0.01
ATP5MC1	mRNA	RNAseq	-1.17	0.01	0.09
ATP5MC3	mRNA	RNAseq	-1.19	0.00	0.02
ATP5MD	mRNA	RNAseq	-1.18	0.00	0.03
ATP5MF	mRNA	RNAseq	-1.11	0.03	0.17
ATP5MF	protein	Proteomics	-1.41	0.13	0.48
ATP5MG	mRNA	RNAseq	-1.11	0.00	0.05
ATP5PB	mRNA	RNAseq	-1.10	0.03	0.17
ATP5PD	mRNA	RNAseq	-1.09	0.04	0.18
ATP5PD	protein	Proteomics	-1.10	0.28	0.64
ATP5PF	mRNA	RNAseq	-1.11	0.03	0.17
ATP5PF	protein	Proteomics	-1.12	0.41	0.73
ATP5PO	mRNA	RNAseq	-1.13	0.01	0.09
ATP5PO	protein	Proteomics	-1.14	0.09	0.43

Fold change is PA compared to control animals. P values cited are nominal values, false discovery rate adjusted Q values were calculated by Benjamini and Hochberg method.

Table S3. Correlation between hepatic ATP levels and OXPHOS genes in adolescent control and prenatally androgenized males.

**OXPHOS**	**Correlation with hepatic ATP**	
**Gene ID**	**Pearson r**	**P value**
NDUFA1	0.31	0.06
NDUFA4	0.38	0.03
NDUFA12	0.27	0.09
NDUFAB1	0.46	0.01
NDUFB3	0.31	0.06
NDUFB4	0.37	0.03
NDUFB10	0.31	0.06
NDUFA9	0.56	0.00
NDUFB5	0.36	0.04
NDUFB11	0.31	0.06
NDUFC1	0.28	0.08
NDUFS4	0.39	0.02
NDUFS7	0.42	0.02
NDUFV2	0.30	0.07
UQCRB	0.27	0.09
UQCRC2	0.37	0.03
UQCRFS1	0.35	0.04
COX5A	0.27	0.09
COX5B	0.33	0.05
COX7B	0.35	0.04
ATP5F1B	0.27	0.09
ATP5MC1	0.32	0.05
ATP5MD	0.32	0.06

ATP5MG	0.36	0.04
ATP5PD	0.39	0.02
ATP5PF	0.30	0.07
ATP5PO	0.40	0.02

Table S4. Differentially expressed genes and proteins relevant to hepatic fatty acid and cholesterol metabolism in adolescent prenatally androgenized males as compared with adolescent male controls.

Gene ID	Template	Detection technique	Fold change	P value	Q Value
**Hepatic FA uptake and trafficking**					
CD36	mRNA	RNAseq	1.26	0.11	0.34
SLC27A5	mRNA	RNAseq	1.11	0.06	0.23
SLC27A5	protein	Proteomics	1.29	0.00	0.12
FABP5	mRNA	RNAseq	1.40	0.04	0.19
**Mitochondrial uptake of FA**					
CPT1A	mRNA	RNAseq	1.33	0.00	0.01
CPT1A	protein	Proteomics	1.22	0.01	0.14
CPT1B	mRNA	RNAseq	1.42	0.00	0.01
SLC25A20	mRNA	RNAseq	1.16	0.09	0.30
CPT2	mRNA	RNAseq	1.15	0.01	0.09
CPT2	protein	Proteomics	1.76	0.10	0.44
CRAT	mRNA	RNAseq	1.17	0.01	0.09
**Fatty acid activation**					
ACSS2	mRNA	RNAseq	-2.03	0.00	0.02
ACSS2	protein	Proteomics	-1.32	0.48	0.79
ACSL3	mRNA	RNAseq	-1.44	0.00	0.01
ACSL5	mRNA	RNAseq	-1.19	0.02	0.11
ACSL5	protein	Proteomics	-1.23	0.00	0.13
**Mitochondrial beta oxidation**					
ACADSB	mRNA	RNAseq	-1.25	0.00	0.05
ACADSB	protein	Proteomics	-1.19	0.02	0.25
ECHS1	mRNA	RNAseq	-1.17	0.01	0.09
ECHS1	protein	Proteomics	-1.16	0.02	0.24
DECR1	mRNA	RNAseq	-1.22	0.00	0.06
DECR1	protein	Proteomics	-1.14	0.07	0.39
**Peroxisomal beta oxidation**					
ACOX2	protein	Proteomics	5.02	0.00	0.08
EHHADH	protein	Proteomics	3.53	0.00	0.10
ACAA1	protein	Proteomics	1.57	0.00	0.07
CROT	mRNA	RNAseq	1.19	0.02	0.11
**Hepatic FA lipogenesis, elongation and desaturation**					
MLYCD	mRNA	RNAseq	1.2	0.00	0.06
ACLY	mRNA	RNAseq	-1.9	0.00	0.00
ACLY	protein	Proteomics	-2.0	0.03	0.29
ACACA	mRNA	RNAseq	-1.8	0.00	0.01
FASN	mRNA	RNAseq	-2.5	0.00	0.03
FASN	protein	Proteomics	-2.5	0.04	0.32
ELOVL6	mRNA	RNAseq	-2.5	0.00	0.00
SCD	mRNA	RNAseq	-4.3	0.00	0.00
**Cholesterol trafficking and metabolism**					
NPC1	mRNA	RNAseq	-1.34	0.00	0.06
NPC2	mRNA	RNAseq	-1.02	0.60	0.81
SOAT2	mRNA	RNAseq	1.2	0.00	0.03
**Lipid droplet accumulation**					
PLIN2	mRNA	RNAseq	1.95	0.01	0.07
PLIN5	mRNA	RNAseq	1.27	0.00	0.03

Fold change is PA compared to control animals. P values cited are nominal values, false discovery rate adjusted Q values were calculated by Benjamini and Hochberg method.

Table S5. Differentially expressed genes and proteins relevant to hepatic glucose metabolism in adolescent prenatally androgenized males as compared with adolescent male controls.

Gene ID	Template	Detection technique	Fold change	P value	Q Value
**Glycolysis**					
HK1	mRNA	RNAseq	1.01	0.87	0.95
HK2	mRNA	RNAseq	-1.09	0.11	0.34
HK3	mRNA	RNAseq	-1.04	0.73	0.88
PFKL	mRNA	RNAseq	-1.09	0.37	0.65
PFKL	protein	Proteomics	-1.28	0.01	0.14
PKLR	mRNA	RNAseq	-1.05	0.43	0.69
PKLR	protein	Proteomics	-1.33	0.03	0.28
**Pyruvate transport**					
MPC1	mRNA	RNAseq	1.26	0.01	0.06
MPC2	mRNA	RNAseq	-1.05	0.34	0.62
**Pyruvate dehydrogenase complex**					
PDHA1	mRNA	RNAseq	-1.09	0.15	0.40
PDHA1	protein	Proteomics	-1.42	0.00	0.08
PDHB	mRNA	RNAseq	-1.10	0.04	0.19
DLAT	mRNA	RNAseq	-1.14	0.00	0.03
DLAT	protein	Proteomics	-1.33	0.03	0.29
**PDHC kinases**					
PDK1	mRNA	RNAseq	1.01	0.88	0.95
PDK2	mRNA	RNAseq	1.04	0.37	0.65
PDK3	mRNA	RNAseq	-1.10	0.17	0.42
PDK4	mRNA	RNAseq	1.55	0.00	0.01
**Gluconeogenesis**					
PC	mRNA	RNAseq	1.82	0.00	0.01
PC	protein	Proteomics	1.17	0.16	0.52
PCK1	mRNA	RNAseq	1.12	0.18	0.44
FBP1	mRNA	RNAseq	1.03	0.73	0.88
FBP1	protein	Proteomics	1.13	0.07	0.38
FBP2	mRNA	RNAseq	1.54	0.01	0.09
G6PC2	mRNA	RNAseq	1.21	0.08	0.28
**Glycogen synthesis**					
GYS2	mRNA	RNAseq	1.21	0.01	0.11

Fold change is PA compared to control animals. P values cited are nominal values, false discovery rate adjusted Q values were calculated by Benjamini and Hochberg method.

Table S6. Correlation between hepatic free cholesterol levels and OXPHOS genes in adolescent control and prenatally androgenized males.

**OXPHOS**	**Correlation with hepatic free cholesterol**	
**Gene ID**	**Pearson r**	**P value**
**Complex I**		
NDUFA1	-0.27	0.16
NDUFA2	-0.16	0.41
NDUFA4	-0.32	0.09
NDUFA5	-0.48	0.01
NDUFA7	-0.28	0.14
NDUFA9	-0.44	0.01
NDUFA12	-0.39	0.03
NDUFAB1	-0.49	0.00
NDUFB1	-0.14	0.47

NDUFB3	-0.42	0.02
NDUFB4	-0.33	0.08
NDUFB5	-0.39	0.04
NDUFB6	-0.39	0.04
NDUFB8	-0.30	0.12
NDUFB9	-0.41	0.03
NDUFB10	-0.42	0.02
NDUFB11	-0.24	0.22
NDUFC1	-0.25	0.20
NDUFC2	-0.29	0.13
NDUFS3	-0.44	0.01
NDUFS4	-0.41	0.02
NDUFS7	-0.15	0.45
NDUFS8	-0.26	0.17
NDUFV2	-0.46	0.01
**Complex III**		
CYC1	-0.31	0.10
CYCS	-0.31	0.10
UQCRB	-0.44	0.02
UQCRC1	-0.30	0.11
UQCRC2	-0.21	0.27
UQCRFS1	-0.33	0.08
UQCRH	-0.32	0.09
**Complex IV**		
MT-CO2	-0.13	0.5
COX5A	-0.39	0.03
COX5B	-0.25	0.21
COX6B1	-0.35	0.07
COX6C	-0.35	0.06
COX7B	-0.42	0.02
**Complex V**		
MT-ATP8	-0.33	0.08
ATP5F1A	-0.29	0.13
ATP5F1B	-0.18	0.34
ATP5F1E	-0.29	0.13
ATP5F1C	-0.35	0.06
ATP5IF1	-0.44	0.01
ATP5MC1	-0.20	0.29
ATP5MC3	-0.28	0.10
ATP5MD	-0.36	0.05
ATP5MF	-0.39	0.03
ATP5MG	-0.45	0.01
ATP5PF	-0.38	0.04
ATP5PD	-0.37	0.05
ATP5PO	-0.39	0.04

Table S7. Differentially expressed genes and proteins relevant to hepatic mitochondrial transporters, translocases and channels in adolescent prenatally androgenized males as compared with adolescent male controls.

Gene ID	Template	Detection technique	Fold change	P value	Q Value
Mitochondrial transporters, translocases and channels					
CLIC2	mRNA	RNAseq	-1.33	0.00	0.05
SFXN3	mRNA	RNAseq	-1.34	0.02	0.12
SLC25A3	mRNA	RNAseq	-1.13	0.00	0.01
SLC25A5	mRNA	RNAseq	-1.28	0.00	0.03

SLC25A11	mRNA	RNAseq	-1.11	0.02	0.13
SLC25A12	mRNA	RNAseq	-1.24	0.01	0.06
SLC25A17	mRNA	RNAseq	-1.10	0.03	0.15
SLC25A25	mRNA	RNAseq	1.31	0.02	0.13
SLC25A29	mRNA	RNAseq	1.39	0.00	0.02
SLC25A30	mRNA	RNAseq	1.95	0.00	0.02
SLC25A32	mRNA	RNAseq	1.11	0.02	0.14
SLC25A34	mRNA	RNAseq	1.52	0.00	0.00
SLC25A35	mRNA	RNAseq	-1.40	0.00	0.01
SLC25A42	mRNA	RNAseq	1.36	0.00	0.01
SLC25A44	mRNA	RNAseq	1.12	0.01	0.10
SLC25A45	mRNA	RNAseq	1.89	0.00	0.02
SLC25A47	mRNA	RNAseq	1.77	0.00	0.03
SLC25A48	mRNA	RNAseq	1.25	0.00	0.02
TIMM8B	mRNA	RNAseq	1.13	0.01	0.10
TIMM9	mRNA	RNAseq	-1.23	0.00	0.01
TIMM21	mRNA	RNAseq	-1.11	0.00	0.05
TIMM23	mRNA	RNAseq	-1.23	0.00	0.01
TIMM50	mRNA	RNAseq	-1.19	0.00	0.01
TIMMDC1	mRNA	RNAseq	-1.15	0.00	0.02
TOMM5	mRNA	RNAseq	-1.15	0.01	0.09
TOMM22	mRNA	RNAseq	-1.16	0.02	0.13
TOMM34	mRNA	RNAseq	-1.21	0.02	0.14
TOMM40	mRNA	RNAseq	-1.21	0.00	0.05
TOMM40	protein	Proteomics	-1.30	0.18	0.54
TOMM70A	mRNA	RNAseq	-1.08	0.02	0.12
VDAC2	mRNA	RNAseq	-1.10	0.01	0.06
VDAC3	mRNA	RNAseq	-1.10	0.01	0.10

Fold change is PA compared to control animals. P values cited are nominal values, false discovery rate adjusted Q values were calculated by Benjamini and Hochberg method.

Table S8. Differentially expressed genes and proteins relevant to hepatic mitochondrial quality control in adolescent prenatally androgenized males as compared with adolescent male controls.

Gene ID	Template	Detection technique	Fold change	P value	Q Value
**Mitochondrial Fusion**					
MFN1	mRNA	RNAseq	1.0	0.57	0.79
MFN2	mRNA	RNAseq	1.1	0.08	0.28
OPA1	mRNA	RNAseq	-1.1	0.03	0.17
OPA1	protein	Proteomics	-1.1	0.50	0.80
**Mitochondrial Fission**					
DNM1L	mRNA	RNAseq	-1.2	0.00	0.03
MTFP1	mRNA	RNAseq	-1.4	0.00	0.05
MTFR1	mRNA	RNAseq	-1.1	0.01	0.07
**Mitophagy**					
CALCOCO2	mRNA	RNAseq	-1.3	0.00	0.00
FUNDC1	mRNA	RNAseq	-1.1	0.00	0.04
GABARAPL2	mRNA	RNAseq	-1.1	0.00	0.01
OPTN	mRNA	RNAseq	-1.1	0.01	0.09
PARK2	mRNA	RNAseq	1.17	0.01	0.07
PARK7	mRNA	RNAseq	-1.16	0.00	0.01
PHB1	mRNA	RNAseq	-1.2	0.00	0.03
PINK1	mRNA	RNAseq	-1.1	0.04	0.19
PGAM5	mRNA	RNAseq	-1.1	0.01	0.07
RHOT1	mRNA	RNAseq	-1.1	0.00	0.03

USP30	mRNA	RNAseq	1.1	0.00	0.01
ZNF746	mRNA	RNAseq	1.1	0.01	0.08

Fold change is PA compared to control animals. P values cited are nominal values, false discovery rate adjusted Q values were calculated by Benjamini and Hochberg method.

Table S9. Correlation between ROS levels and hepatic lipid metabolism, detoxification potential and fibrosis effectors in in adolescent control and prenatally androgenized males.

**Correlation with hepatic ROS levels**	**Pearson r**	**P value**
**Hepatic cholesterol metabolism**		
Hepatic *CYP7A1* expression	-0.42	0.03
Hepatic *NPC1* expression	-0.41	0.04
Hepatic free cholesterol	0.36	0.06
**Mitochondrial FA uptake**		
Hepatic *CPT1A* expression	0.54	0.00
Hepatic *CPT1B* expression	0.64	0.00
**Regulation of lipid metabolism**		
Hepatic *PPARA* expression	0.40	0.04
Hepatic *NR1I2* expression	0.69	0.00
**Liver detoxification potential**		
Hepatic *GSS* expression	-0.38	0.05
Hepatic *GSTM1* expression	-0.36	0.07
Hepatic *GSTO1* expression	-0.51	0.01
Hepatic *GPX4* expression	-0.43	0.02
Hepatic GPX7 expression	0.36	0.06
**Fibrosis effectors**		
Hepatic *FGFR2* expression	0.47	0.01
Hepatic *FGFR3* expression	0.42	0.03
Hepatic *COL4A1 expression*	0.51	0.01
Hepatic COL4A2 expression	0.52	0.01
Hepatic *COL4A4* expression	0.33	0.09
Hepatic *COL18A1 expression*	0.66	0.00
Hepatic *COL27A1* expression	0.47	0.01
